# 

**Published:** 1890-08

**Authors:** 


### BOOKS AND PAMPHLETS RECEIVED.

Das thierarztliche Unterrichtswesen Deutschlands in seiner geschichtlichen Entwickelung und Bedentung fur den thierarztlichen stand. Ein Gedenkblatt aus Anlass der feier des 100 jahrigen Bestehens der thierarztlichen Hochschule zu Berlin bearbeitet von Dr. Georg Schneidemuhl, Privatdocent an der Universitat in Kiel. Leipzig. Arthur Felix. 1890.

Festchrift zur Feier des funfundzwanzigjahrigen Bestehens des naturwissenschaftlichen Vereins zu Bremen.

Vierteljahrschrift der Naturforschenden Gesellschaft in Zurich. 1888–89.

Archives Neerlandaises des Sciences Exactes et Naturelles. Tome xxiv. Liv. 2 and 3.

Annales et Bulletin de la Societé de Médecine de Gand. 1890.

Bulletin mensuel Sociéte Linneenne du Nord de la France. Sept. and October, 1889.

Bulletin de la Société Zoologique. Paris.

Bulletin de L'Academie Royale de Médecine de Belgique. Bruxelles, 1890.

Proceedings and Transactions of the Natural History Society of Glasgow. Vol. II, Part 2, 1887–88; Vol. III, Part 1, 1888–89.

Proceedings of the Canadian Institute. Vol. VII, Fas. 2. Toronto, 1890.

Transactions Kansas Academy of Science. Vol. XII, part 1, 1889.

Annual Report of the Zoological and Acclimatization Society of Victoria. Melbourne, 1890.

Verslag van den toestand van het Koninklijk zoologisch-Botanesch Genootschap te S'Gravenhage over het jaar 1889.

Fuhrer durch den zoologischen Garten zu Breslau, von H. Stechmann, Dîrektor des Gartens. Festausgabe zur Feier des 25 jahrigen Bestehens.

Johns Hopkins University Circulars. Baltimore.

Ottawa Naturalist.

American Naturalist. Philadelphia.

Il Naturalista Siciliano. Palermo.

